# Epidemiology and molecular characterization of lumpy skin disease virus in cattle in the Poro Region of Ivory Coast

**DOI:** 10.3389/fvets.2026.1759378

**Published:** 2026-03-06

**Authors:** Mireille Catherine Kadja, Aristide Anicet Zobo, Edmond Onidje, Laibané Dieudonné Dahourou, Yemienguigna Nougnon Coulibaly, Cyprien Bokpe Yapi, Souahibou Sourokou Sabi, Benjamin Obukowho Emikpe, Irene Kasindi Meki, Tirumala Bharani Kumar Settypalli, William George Dundon, Viskam Wijewardana, Charles Euloge Lamien

**Affiliations:** 1 Ecole Inter-Etats des Sciences et Médecine Vétérinaires de Dakar, Dakar, Senegal; 2 Central Veterinary Laboratory, Bingerville, Côte d'Ivoire; 3 Department of Veterinary Medicine, University of Perugia, Perugia, Italy; 4 Institut des Sciences de l’Environnement et du Développement Rural, Université Daniel Ouezzin Coulibaly, Dédougou, Burkina Faso; 5 School of Veterinary Medicine, Kwame Nkrumah University of Science and Technology, Kumasi, Ghana; 6 Animal Production and Health Laboratory, Joint FAO/IAEA Centre of Nuclear Techniques in Food and Agriculture, Department of Nuclear Sciences and Applications, International Atomic Energy Agency, Vienna, Austria

**Keywords:** epidemiology, Ivory Coast, lumpy skin disease, molecular surveillance, risk factors

## Abstract

**Introduction:**

Lumpy skin disease (LSD) threatens cattle health and productivity in Ivory Coast, where limited resources for livestock management hinder disease control. Moreover, the lack of studies on its prevalence and genetic profile leaves critical gaps in understanding its epidemiology and local risk factors. This study addresses these gaps by investigating LSD viruses’ prevalence, its molecular characteristic and the associated risk factors among cattle in the Poro Region of northern Ivory Coast.

**Methods:**

Using a cross-sectional design, nodule and nasal swab samples were collected from 405 cattle across 36 villages between September 2023 and December 2024 based on syndromic surveillance. The samples were analyzed PCR to confirm LSD virus presence, followed by sequencing of four viral genes: RPO30, GPCR, EEV glycoprotein, and B22R.

**Results:**

Overall, LSD prevalence among cattle showing pox-like lesions and clinical symptoms was found to be 51.85% and varied significantly across localities, reaching 66.67% in M’bengué and 70.87% in Dikodougou. Larger herds (over 50 cattle) had a higher prevalence (76.51%) compared to smaller herds (34.72%), and transhumant herds showed increased prevalence (*p* < 0.001). No significant associations were identified between sex, age, or breed. Phylogenetic analysis indicated that the Ivory Coast LSDV strains clusters with other African field strains, distinct from South-East Asian and Russian recombinants.

**Discussion:**

The present study shows a notable regional difference in the prevalence of LSD in cattle in Ivory Coast, with big and transhumant herds having a higher prevalence rate making the herd size and movement a major risk factor. Molecular analysis demonstrated that Ivory Coast LSD strains are in the same group with other strains found in the African field, indicating that it is necessary to take control measures within the region and provide further surveillance.

## 1 Introduction

Livestock farming is a cornerstone of life across Africa, particularly in West Africa, where it plays a vital role in agricultural production, food security, and nutrition (1, 2). For millions of West Africans, livestock serves as a primary source of food, a foundation for financial stability, a form of investment, and a reserve of wealth, while also holding social and cultural significance. It’s integration into cultural practices and rituals, provides an essential safety net during crises, supporting the livelihoods and resilience of millions (3).

Africa’s livestock sector contributes significantly to the continent’s economy, accounting for 30 to 80% of its agricultural GDP. Globally, nearly 85% of livestock farmers are based in sub-Saharan Africa, making the region a global leader in terms of livestock reliance. However, it produces only 2.8% of the world’s meat and milk, despite housing over 14% of global animal resources, highlighting its vast untapped potential (4). As population and urbanization growth increase, so does the demand for animal products, particularly in West Africa, where countries are divided into consumer and production hubs. Coastal nations serve as primary consumer markets, while Sahelian countries act as central production centers (5).

Despite this demand, Africa’s livestock potential remains largely underexploited due to inadequate policies that lead to structural imbalances. In Ivory Coast, a nation with a strong agricultural base, livestock remains a secondary industry. The production of meat and dairy products meets only a fraction of national needs, with deficits of 55.4 and 87.4%, respectively (6, 7). Although the Ivorian government has implemented initiatives to address these shortages, certain livestock sectors still face substantial challenges in meeting demand. For example, the cattle sector continues to struggle with competitiveness, a key hurdle in the fight against rural poverty (8). Moreover, the sector faces dietary and health-related constraints that limit growth and productivity, exacerbated in recent years by climate change, which has increasingly impaired productive capacity (9).

Among health-related issues, viral diseases present some of the most significant obstacles, given their potential to spread swiftly across herds, severely limiting productivity. In Ivory Coast, several bovine diseases are endemic, including bovine tuberculosis, foot-and-mouth disease, contagious bovine pleuropneumonia, animal trypanosomiasis, and lumpy skin disease. Lumpy skin disease (LSD), caused by LSD virus (LSDV) (recently renamed *Capripoxvirus lumpyskinpox*), a member of *Capripoxvirus* genus within the Poxviridae family, particularly affects cattle and buffalo and is a notifiable disease with strict reporting requirements (10, 11). LSD causes skin nodules leading to substantial economic losses through decreased milk production, weight loss, higher abortion rates, and a deterioration in wool and hide quality, ultimately increasing vulnerability to other infections, which can be fatal (12). Since the year 2000, lumpy skin disease (LSD), originally confined to sub-Saharan Africa, has expanded north- and eastwards into the Middle East, Central Asia, and South and Southeast Asia (13). In North Africa, countries other than Egypt remained LSD-free until its first detection in Libya in 2023, followed by Algeria and Tunisia in 2024 (14). As of 2025, LSD has re-emerged in Europe for the first time since the 2015–2018 epidemic, with confirmed outbreaks in Italy and France in June 2025 (15), and Spain in October 2025.

Although LSD is endemic for several decades in Ivory Coast, little is known on the epidemiology. A comprehensive understanding of the epidemiology of LSD is essential for developing targeted control strategies. Furthermore, studies utilizing molecular techniques to investigate the prevalence and associated risk factors of this disease in cattle populations in Ivory Coast are scarce, despite its significant impact on livestock productivity and rural economies. This study aims to address this limitation by employing molecular techniques to characterize the epidemiology of LSDV, focusing on its prevalence and identifying specific risk factors among cattle in the Poro Region of Ivory Coast.

## 2 Materials and methods

### 2.1 Study area

**Figure 1:**
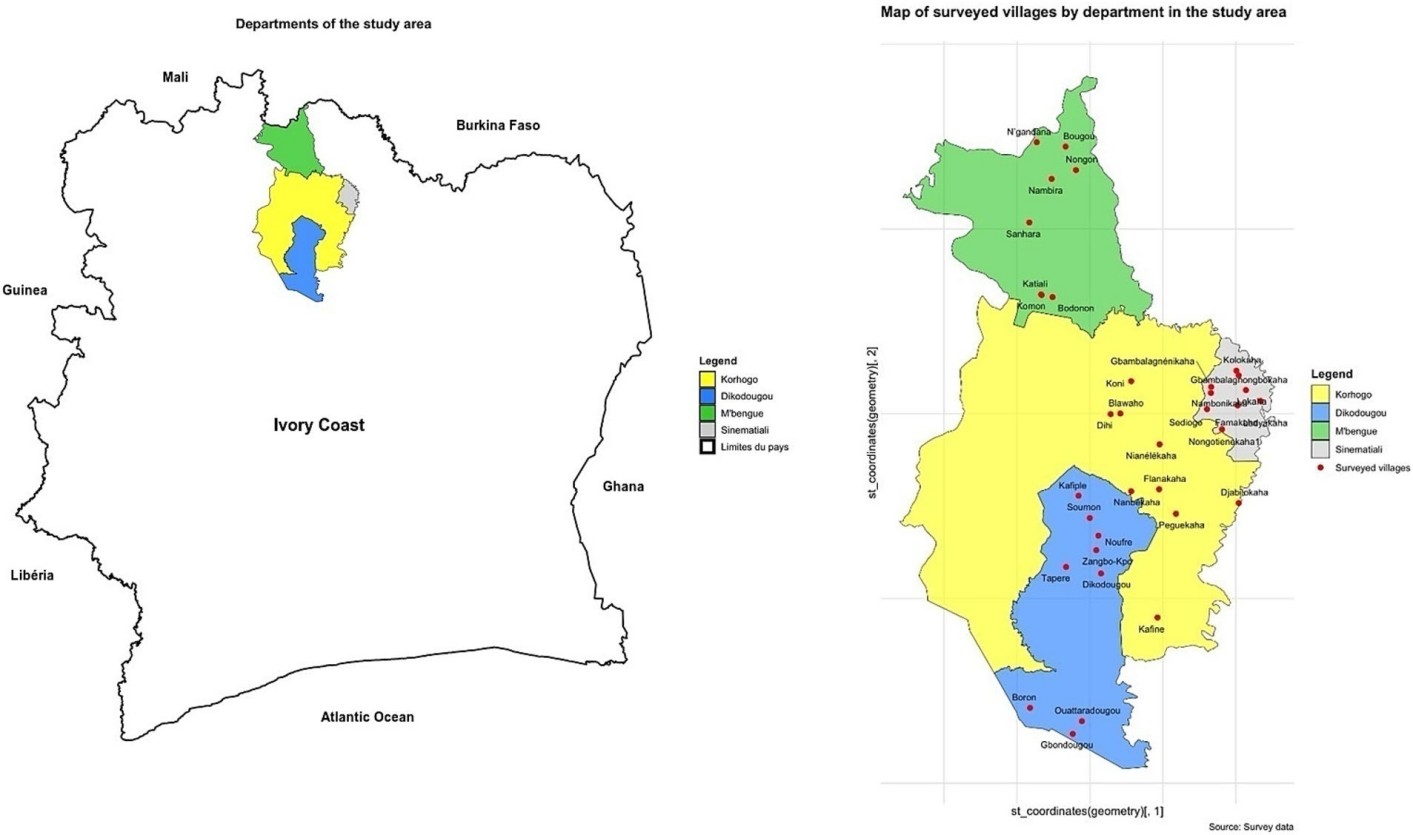
Map of the study area.

The present study was conducted in the Poro Region, located in northern Ivory Coast between latitudes 8°26 and 10°27 North and longitudes 5°17 and 6°19 West (Figure 1). This region, bordered by Mali to the north, Béré to the south, Tchologo and Hambol to the east, and Bagoué to the west, spans approximately 13,400 km^2^ and is home to an estimated population of 1,040,500, as recorded in the 2021 national census. Korhogo serves as the regional capital, with administrative divisions comprising four departments (Dikodougou, Korhogo, M’bengué, and Sinématiali), 27 sub-prefectures, 11 municipalities, and roughly 1,000 villages (16). The climate alternates between a dry season, influenced by harmattan winds from December to January, and a rainy season from May to October, with peak rainfall in July and August. Vegetation in the area is characteristic of tree-dotted savanna, where trees and shrubs reach heights of 8 to 12 meters creating an environment favorable for ruminants. The Poro Region’s economy is largely agropastoral, with agriculture and livestock representing 44% of its economic activities. Its success in livestock and complementary industries, such as fishing, mining, commerce, tourism, and industry, reflects its strong economic design for supporting cattle and other ruminant populations (17). Given these favorable conditions, resources and its high density of domesticated ruminant livestock, Poro Region was selected as the study site for the present research.

### 2.2 Study design

A cross-sectional study was conducted between September 2023 and December 2024 to determine the prevalence, geographic distribution, and associated risk factors of LSD in cattle within the Poro Region of Ivory Coast. Fieldwork was carried out in 36 villages across 12 sub-prefectures, selected through stratified random sampling within the four departments of the region. In each village, a minimum of 11 cattle were sampled, with animals showing clinical signs of LSD prioritized.

Data on potential LSD transmission risk factors were collected through a structured questionnaire, designed in Kobotoolbox (2019) and administered to livestock owners. The questionnaire gathered information on clinical signs, herd size and composition, watering and feeding practices, and the seasonal occurrence of the disease. In total, 101 questionnaires were completed throughout the region, providing insight into possible risk factors associated with LSD transmission.

### 2.3 Sample size determination

The sample size for this study was calculated using Cochran’s formula, which considers the desired confidence level, population proportion, and margin of error to ensure statistical precision (18):

$$n=\frac{Z^{2}.p.(1-p)}{d^{2}}$$

For this study, a 95% confidence level was selected, corresponding to a Z-score of 1.96. The margin of error was set at 0.05 (±5%), and an expected population proportion (*p*) of 0.5 was used. This proportion was chosen to maximize the sample size and account for the absence of specific prevalence data for LSD within the target population, providing a conservative and reliable estimate. Using these parameters, the required sample size was 384. However, in this study, 405 animals showing clinical signs of LSD were sampled, using syndromic surveillance. Among these, 15 animals were sampled twice, once for a nasal swab and once for a skin nodule, resulting in a total of 420 samples. The relatively small quantity of skin nodule samples is due to the requirement for farmer consent, as collection of skin nodules involves excision of lesion tissue, which can cause discomfort to the animals. Most farmers did not agree to the collection of skin nodule samples for this reason. Additionally, a total of 101 questionnaires were completed across the region.

### 2.4 Sample collection and processing

Nodule or nasal swab samples were collected from each animal, placed in viral transport medium and labeled, before being transported under cold chain conditions to the Korhogo Veterinary Laboratory, where initial preservation at −20 °C was performed. Subsequently, all samples were transferred to the Central Veterinary Laboratory (CVL) in Bingerville, for further processing. At CVL, 250 μL aliquots were prepared in Eppendorf tubes and stored at −80 °C until analysis. Viral DNA extraction was performed in three steps using the QIAamp^®^ DNA Mini Kit: lysis (with proteinase K, Buffer AL, and ethanol added to each sample), washing, and elution. The extracted DNA was subsequently stored at −20 °C until molecular analysis was conducted to confirm the presence of the LSDV.

### 2.5 Detection of *Capripoxvirus* genome

Real time PCR to detect LSDV genome in cattle samples, was performed following Bowden et al. (26) method with slight modifications. Briefly, the reaction mix was prepared using the iQ^™^ Supermix kit (Bio-Rad, United States), incorporating LSD-specific primers CaPV074F1 (forward primer; AAAACGGTATATGGAATA GAGTTGGAA) and CaPV074R1 (reverse primer; AAATGAAAC CAATGGATGGGATA) along with the FAM-labelled probe CaPV074P1 (FAM-TGGCTCATAGATTTCCT-MGB/NFQ) for fluorescent detection. The reaction mix was distributed into a 96-well PCR plate, with each well containing 20 μL of reaction mix and 5 μL of sample. Positive and negative controls were included to ensure accuracy and detect any potential contamination. Amplification was carried out on a QuantStudio PCR machine, using a thermal cycling program that consisted of an initial denaturation at 95 °C for 10 min, followed by 45 cycles of denaturation at 95 °C for 15 s, annealing at 60 °C for 15 s, and extension at 72 °C for 60 s. A positive result for LSD was indicated by a sigmoidal amplification curve with a Cq value < 35.

### 2.6 Sequencing analysis

To characterize the genetic profile of the Ivory Coast LSDV isolates, sequencing was performed on four key *Capripoxvirus* (CaPV) gene targets: RNA polymerase 30 kDa subunit (RPO30), G protein-coupled receptor (GPCR), extracellular enveloped virus (EEV) glycoprotein, and the CaPV homolog of the variola virus B22R gene. Five skin nodule samples where all targeted genes were successfully amplified were selected for Sanger sequencing at LGC Genomics (Germany). None of the nasal swab samples produced successful amplification, likely due to their low viral load, which did not allow for reliable sequencing. Sequencing was performed in both directions to ensure high-quality consensus sequences. The selected samples represented both the 2023 and 2024 outbreaks, providing a snapshot of the circulating LSDV strains.

Representative CaPV sequences were retrieved from GenBank, and multiple sequence alignments were performed for each targeted gene. Phylogenetic analyses were conducted using the complete RPO30 and GPCR gene sequences. A neighbor-joining tree for the RPO30 gene and a maximum-likelihood tree for the GPCR gene, were con-structed applying the Tamura-Nei model and Gamma distribution (16–18). These phylogenetic trees enabled assessment of the evolutionary relationships of the Ivory Coast LSDV isolates with other known LSDV strains.

For additional comparative analysis, sequence similarities and structural variations in the multiple sequence alignments of the EEV glycoprotein and B22R partial gene sequences, together with Ivory Coast isolates were evaluated. Identical nucleotides were marked, with regions of variation, such as insertions or deletions, highlighted for comparative analysis.

### 2.7 Data analysis

Data obtained from field surveys and PCR tests were entered and recorded in Microsoft Excel 2016. The prevalence of LSD was calculated by dividing the number of positive nodule or swab samples by the total number of samples tested. A 95% confidence interval (CI) was calculated for each prevalence value using the formula for simple random sampling: $\mathrm{CI}=p\pm 1,96\times \sqrt{\frac{\left(p(1-p)\right)}{N}}$ where *p* represents the prevalence and *N* is the sample size.

Descriptive and analytical statistical analyses, including chi-square tests and odds ratios, were conducted using R software (version 3.5.2) through the RStudio interface. To identify factors associated with LSD infection, univariate logistic regression was performed to determine unadjusted odds ratios. Variables with a *p*-value ≤ 0.20 from this analysis were then included in a multivariate analysis using a logistic regression model. The identification of associated factors was based on examining odds ratios, their confidence intervals, and corresponding *p*-values. The significance threshold for these analyses was set at 0.05.

## 3 Results

### 3.1 Herd characteristics and management systems in the Poro Region

The sampled herds displayed a range of characteristics (Table 1): 73.27% consisted solely of cattle, while 26.73% were mixed herds, all managed under an extensive farming system. Herd sizes varied, with 59.41% containing 50 or fewer animals and 40.59% holding more than 50. Transhumance was uncommon, practiced by only 10.89% of the herds, while 89.11% did not engage in this practice. The breed composition included primarily crossbreeds (49.63%), followed by Zebu (27.16%), Ndama (22.72%), and a small portion of Gudali (0.49%). Females were the majority at 70.37%, with males accounting for 29.63%. In terms of age, 46.67% of cattle were younger than 2 years, while 53.33% were older than 2 years.

**Table 1:** Characteristics of sampled herds.

Variables	Category	Count (*n*)	Percentage (%)
Herd type	Mixed	27	26.73
	Cattle only	74	73.27
Farming system	Extensive	100	100.00
	Intensive	0	0.00
Herd size	≤ 50	60	59.41
	> 50	41	40.59
Transhumance	Yes	11	10.89
	No	90	89.11
Breed	Zebu	110	27.16
	Ndama	92	22.72
	Gudali	2	0.49
	Crossbreed	201	49.63
Sex	Female	285	70.37
	Male	120	29.63
Age	< 2 years	189	46.67
	> 2 years	216	53.33
Vaccination	Yes	0	0.00
	No	405	100.00

### 3.2 Prevalence of LSD in the Poro Region

**Figure 2:**
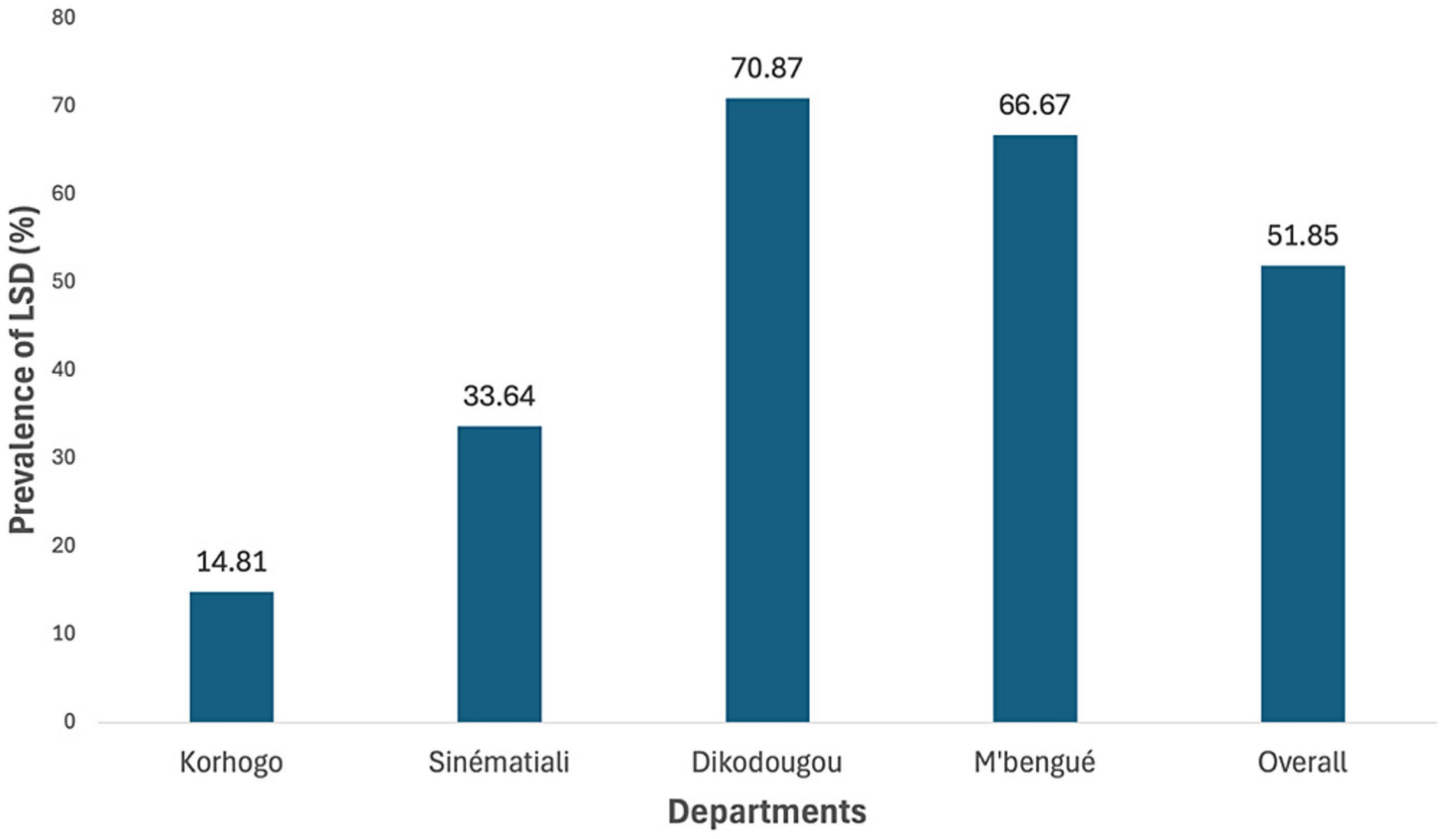
Prevalence of LSD in cattle by department in the Poro Region.

The prevalence of LSD among cattle in the Poro Region between September 2023 and December 2024 was determined to be 51.85%, with 210 nasal swabs out of 405 samples testing positive (Figure 2). All 15 skin nodule samples were positive, confirming the positivity of the corresponding nasal swabs from the same animals. Prevalence varied significantly across localities, as shown in Table 2. The highest rates were observed in the departments of M’bengué and Dikodougou, with prevalence rates of 66.67 and 70.87%, respectively. In contrast, the department of Korhogo recorded the lowest prevalence at 14.81%.

**Table 2:** Variation of LSD prevalence based on different factors in the Poro Region, Ivory Coast.

Variables	Factors	Number of samples	Number of positives (%)	*p* value
Herd type	Mixed	109	48 (44.04)	0.07218
	Cattle Only	296	162 (54.73)	
Herd size	≤ 50	239	83 (34.72)	2.97e-16
	> 50	166	127 (76.51)	
Transhumance	Yes	45	39 (86.66)	4.52e-07
	No	360	171 (47.5)	
Sex	Female	285	149 (52.28)	0.875
	Male	120	61 (50.83)	
Age group	< 2 years	189	96 (50.79)	0.6875
	> 2 years	216	114 (52.78)	
Breed	Zebu	110	53 (48.18)	0.678
	Ndama	92	47 (51.09)	
	Gudali	2	1 (50.00)	
	Crossbreed	201	109 (54.23)	

### 3.3 Factors influencing LSD prevalence in the Poro Region, Ivory Coast

The variability in LSD prevalence according to different factors in the Poro Region is summarized in Table 2. A slightly higher prevalence was observed in cattle-only herds (54.73%) compared to mixed herds (44.04%), though this difference was not statistically significant (*p* = 0.07218). Herd size significantly influenced prevalence, with larger herds (>50) showing a higher rate (76.51%) than smaller herds (≤50), which had a prevalence of 34.72% (*p* = 2.97e-16). Transhumant herds exhibited a significantly higher LSD prevalence (86.66%) compared to non-transhumant herds (47.5%) (*p* = 4.52e-07). No significant differences in LSD prevalence were found based on sex (*p* = 0.875), age group (*p* = 0.6875), or breed (*p* = 0.678).

#### 3.3.1 Statistical analysis of LSD risk factors among cattle of Poro Region, Ivory Coast

Univariate logistic regression analysis (Table 3) revealed that herd type was not significantly associated with LSD infection with odds ratio (OR) of 0.65 (95% CI: 0.42–1.01) for mixed herds compared to cattle-only herds (*p* = 0.0569). Herds with more than 50 cattle had a significantly higher odds of infection (OR = 6.12, 95% CI: 3.94–9.65, *p* = 1.92E-15) compared to those with 50 or fewer. Transhumant herds had significantly increased odds of infection (OR = 7.00, 95% CI: 3.10–18.79, *p* = 1.65E-05) compared to non-transhumant herds. No significant associations were found for sex (male OR = 0.94, 95% CI: 0.61–1.45, *p* = 0.79), age group (>2 years OR = 1.105, 95% CI: 0.75–1.63, *p* = 0.616), or breed, with Zebu, Ndama, and Crossbreed all showing non-significant ORs compared to the Gudali reference.

**Table 3:** Univariate and multivariate logistic regression analysis of factors associated with LSD virus infection.

Variables	Factors	Univariate OR (95% CI)	Univariate *p* value	Multivariate OR (95% CI)	Multivariate *p* value
Herd type	Cattle only	Ref	-	Ref	-
	Mixed	0.65 (0.42–1.01)	0.0569	0.27 (0.13–0.50)	**7.63E-05**
Herd size	≤ 50	Ref	-	Ref	-
	> 50	6.12 (3.94–9.65)	**1.92E-15**	19.8 (10.1–42)	**1.2E-16**
Transhumance	No	Ref	-	Ref	-
	Yes	7.00 (3.10–18.79)	**1.65E-05**	5.2 (2.09–15)	**0.000874**
Sex	Female	Ref	-	Ref	-
	Male	0.94 (0.61–1.45)	0.79	-	-
Age group	< 2 years	Ref	-	-	-
	> 2 years	1.105 (0.75–1.63)	0.616	-	-
Breed	Gudali	Ref		-	-
	Zebu	0.81 (0.03–21.01)	0.886	-	-
	Ndama	1.04 (0.04–26.94)	0.976	-	-
	Crossbreed	1.18(0.046–30.24)	0.905	-	-

Bold values indicate statistically significant associations (*p* < 0.05).

The multivariate logistic regression analysis (Table 3) showed that mixed herds had a significantly lower odds of infection (OR = 0.27, 95% CI: 0.13–0.50, *p* = 7.63E-05) compared to cattle-only herds. Herds with more than 50 animals demonstrated a much higher odds of infection (OR = 19.8, 95% CI: 10.1–42, *p* = 1.2E-16) compared to those with 50 or fewer. Additionally, transhumant herds had significantly greater odds of infection (OR = 5.2, 95% CI: 2.09–15, *p* = 0.000874) compared to non-transhumant herds.

### 3.4 Molecular characteristics of LSDV isolates from Poro Region of Ivory Coast

**Figure 3:**
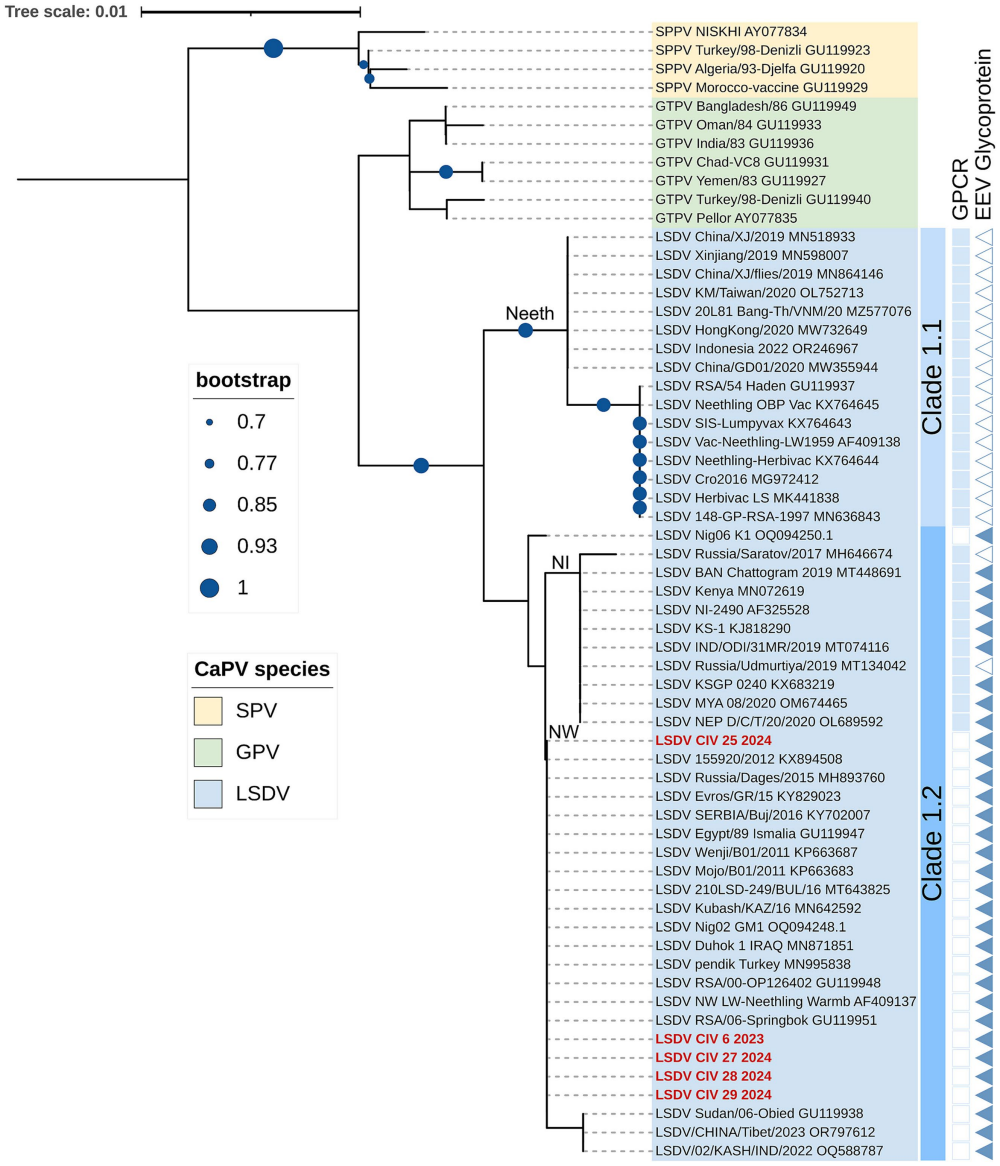
Neighbor-joining tree based on complete RPO30 gene sequences of CaPVs visualized on iTOL, along with LSDV clustering based on the presence (filled box) or absence (empty box) of sequence insertion in the GPCR and the EEV glycoprotein genes. The evolutionary distances were calculated using the Tamura-Nei method. Ivory Coast LSDV isolates are in red.

The RPO30, GPCR, EEV glycoprotein, and the B22R genes were successfully amplified and sequenced from one 2023 sample and four samples collected in 2024, all of which originated from skin nodules and none from nasal swabs. The resulting sequences for each gene were deposited in GenBank under accession numbers PX459670-PX459689. Phylogenetic analysis of the RPO30 gene sequences revealed that all five Ivory Coast LSDV isolates clustered within LSDV Clade I.2. Within this clade, the isolates grouped with Neethling Warmbaths (NW)-like strains, which includes commonly circulating field isolates from Africa [LSDV Nig02 (OQ094248)], the Middle East [LSDV Egypt/89_Ismalia (GU119947)], and Europe [LSDV SERBIA/Buj/2016 (KY702007)] (Figure 3).

**Figure 4:**
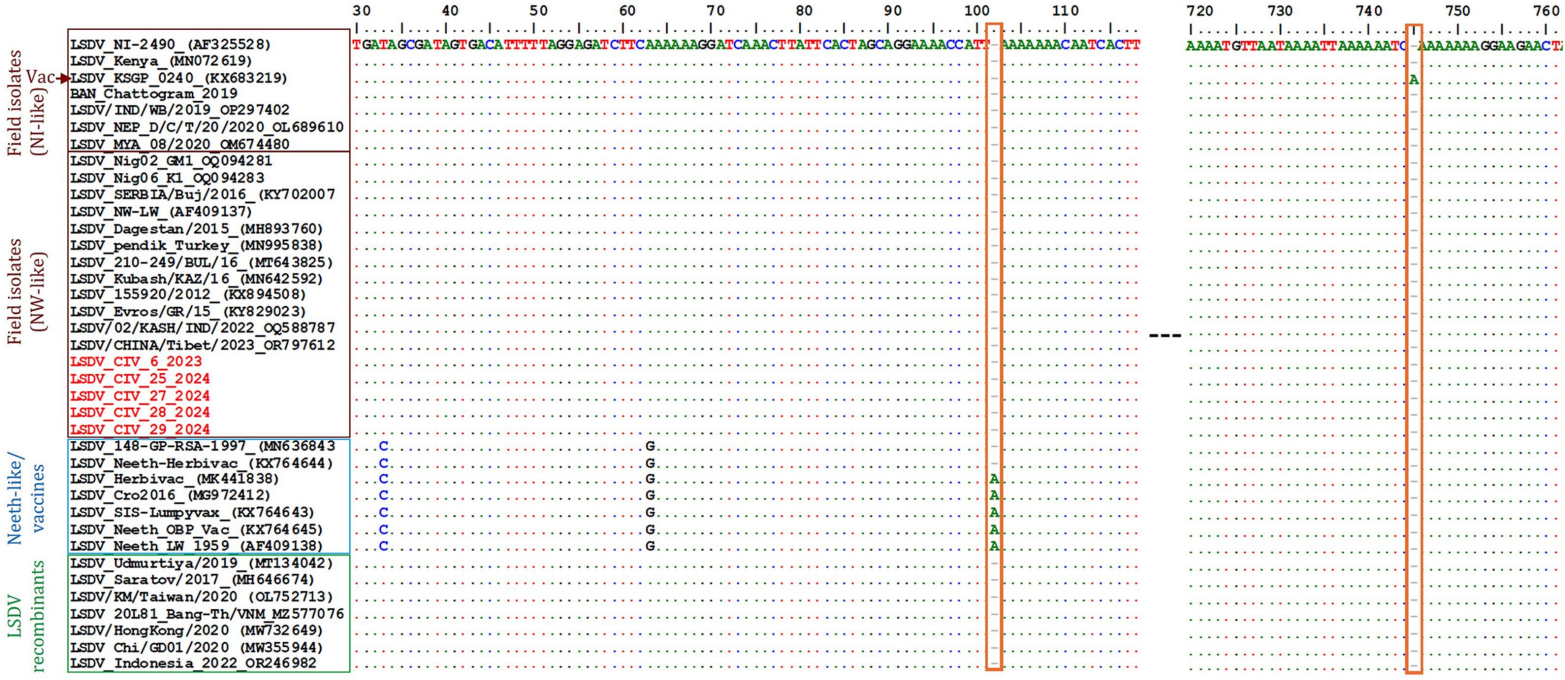
Multiple sequence alignment of the partial B22R gene nucleotide sequences. The Ivory Coast isolates (in red) were aligned with representative LSDV sequences retrieved from GenBank. The nucleotide insertion in LSDV Neethling and LSDV KSGPO-240 vaccines that are absent in the Ivory Coast isolates is shown in the blocks. The dots indicate identical nucleotides in the alignment.

Further phylogenetic analysis based on the GPCR gene confirmed the clustering of the five Ivory Coast LSDV isolates from within Clade 1.2 (Supplementary Figure S1). Sequence alignment of the GPCR gene showed a 12-nucleotide deletion in the Ivory Coast LSDV isolates, a characteristic shared with other NW-like LSDV field isolates (Figure 3). Multiple sequence alignment of the partial EEV glycoprotein gene showed that the Ivory Coast LSDV isolates (in red) lack the 27-nucleotide deletion (175–201) found in Clade 1.1 and recombinant LSDV isolates (Figure 3; Supplementary Figure S2). Lastly, comparative sequence analysis of the B22R sequences demonstrated that the Ivory Coast LSDV isolates (highlighted in red) do not possess the nucleotide insertions present in the LSDV Neethling and LSDV KSGP-0240 vaccine strains at positions 102 and 745, respectively (Figure 4). Collectively, the molecular characterization of these four targeted LSDV genes supports the classification of the Ivory Coast isolates as NW-like LSDV field strains within Clade 1.2, consistent with other circulating LSDV strains in Africa, the Middle East, and Europe.

## 4 Discussion

The present study investigated the prevalence and molecular characteristics of LSD in cattle herds across the Poro Region of Ivory Coast, aiming to understand factors influencing the spread of this disease. The high prevalence of LSD (51.85%, testing positive) among the sampled cattle, underscores the significant health burden LSD poses to the livestock sector in the region. These findings highlight the endemic nature of LSD in the Poro Region, suggesting that the virus is well established and continually circulating within cattle populations. Similar studies in other parts of Africa, such as those by Abebaw (19) and Dubie et al. (20) in Ethiopia, revealed high prevalence rates in regions with minimal control measures, underscoring LSD’s persistence in areas with vulnerable livestock management systems.

A notable finding of this study is the variation in LSD prevalence based on herd size, with herds over 50 animals showing a 76.51% infection rate compared to 34.72% in smaller herds. This aligns with findings from Nasser Faris et al. (10) and Zeedan et al. (21), who reported that, in Egypt, larger herd sizes were associated with increased transmission risk due to higher animal density and challenges in implementing effective biosecurity measures. Dubie et al. (20) observed similar trends in Ethiopia, underscoring the heightened transmission risk in densely packed livestock settings and suggesting that targeted biosecurity interventions in larger herds could mitigate spread (20).

Transhumance was also identified as a critical factor, with transhumant herds showing an infection rate of 86.66% compared to 47.5% in non-transhumant herds. This elevated prevalence aligns with studies by Abebaw (19) and Ochwo et al. (22), where herd mobility increased LSD exposure due to encounters with infected areas. Ochwo et al. (22) emphasized that frequent contact with communal water sources and grazing spaces, particularly in mobile populations, significantly heightens the risk. These findings suggest that improved vector control and tailored vaccination programs are essential for transhumant herds.

Interestingly, factors such as breed, sex, and age did not significantly influence infection rates, with crossbreeds and local breeds displaying similar levels of infection. This suggests that environmental and management factors are often more influential than genetics, consistent with findings by Ochwo et al. (22) and Abebaw (19). This uniform infection rate supports the idea of extensive environmental exposure and transmission across demographic groups, as also observed by Dubie et al. (20) in Ethiopia. However, this contrasts with some studies that have indicated breed-specific vulnerabilities (23, 24).

A notable gap in this study was the lack of vaccination among sampled cattle. Vaccination has proven effective in lowering LSD prevalence, as shown by Nasser Faris et al. (10) and Ezzeldin et al. (25). Implementing a targeted vaccination program, particularly for high-risk groups such as large and transhumant herds, could substantially reduce LSD rates. Collaborative efforts among local authorities, livestock owners, and international agencies are critical to improve vaccine access and administration. Awareness campaigns and biosecurity training, as recommended by Dubie et al. (20), could also play a role in reducing transmission, particularly in high-risk areas.

The genetic analysis of the LSDV isolates from Ivory Coast revealed clustering with NW-like field strains circulating across Africa and the Middle East, rather than vaccine-derived strains. This finding aligns with studies by Dubie et al. (20) in Egypt and Zeedan et al. (21), which highlight that region-specific strains often dominate in endemic areas.

Molecular surveillance techniques, such as those emphasized by Ochwo et al. (22) and Zeedan et al. (21), reveal that genetic markers particularly deletions or mutations in key genes like GPCR, RPO30, and EEV glycoprotein are instrumental in tracking strain lineage and understanding transmission dynamics. The absence of vaccine-associated genetic markers in Ivory Coast isolates, such as those found in attenuated strains, reinforces the need for a tailored vaccination approach that targets the genetic profile of locally circulating field strains. As highlighted by Dubie et al. (20), genetic characterization of field strains is critical for determining effective vaccine efficacy, especially in regions where vaccine failure has been suspected due to strain mismatch. Genetic consistency among LSDV field strains also highlights the adaptability of the virus in diverse ecological settings. Studies like Ezzeldin et al. (25) notes that genetically stable strains across regions reflect minimal mutation accumulation, allowing the virus to thrive across various hosts and environmental conditions without losing virulence. This genetic stability suggests that LSDV has reached a balanced adaptation with its hosts, underscoring the necessity for molecular surveillance and genomic sequencing in monitoring potential genetic drift or recombination events that could influence transmission or vaccine efficacy.

The findings from Ivory Coast thus underscore the importance of a robust molecular surveillance framework that continuously monitors genetic variations within LSDV. Such frameworks enable targeted control strategies, including vaccines specifically designed to match local strains, enhancing the effectiveness of vaccination programs and supporting long-term disease management efforts in endemic regions.

### 4.1 Study limitations

While this study offers valuable insights into LSD prevalence and molecular characteristics, its cross-sectional design limits the ability to draw conclusions about the long-term trends in LSD prevalence or the effectiveness of potential interventions. Future research should adopt a longitudinal approach, to evaluate seasonal changes in disease prevalence and the impact of introduced biosecurity and vaccination practices.

## 5 Conclusion

The findings from this study reveal a high prevalence of LSD in cattle herds across the Poro Region of Ivory Coast, highlighting the disease’s significant impact on livestock health in endemic settings. Larger herd sizes and transhumant practices were found to be key factors associated with higher infection rates, emphasizing the role of herd management in disease transmission. Genetic analysis confirmed that the circulating strain in Ivory Coast aligns with other naturally occurring field strains in Africa, reinforcing the need for region-specific vaccines. The lack of vaccination among sampled herds reveals a critical gap in current disease control strategies. To mitigate the impact of LSD, a targeted approach is recommended, prioritizing vaccination programs with vaccines tailored to local strains, especially for larger and transhumant herds at higher risk. Key biosecurity measures, including the isolation of symptomatic animals, vector control, and improved sanitation in densely populated herds, should be implemented. Training livestock owners in vaccination and biosecurity practices is essential to ensure adherence. Finally, a molecular surveillance system should be established to monitor LSDV genetic variations, and enable early outbreak detection and responsive vaccination strategies.

## Data Availability

The data presented in the study are deposited in the GenBank repository (https://www.ncbi.nlm.nih.gov/genbank/), accession numbers PX459670 to PX459689.
