# Supplementary material for: Epidemiology and molecular characterization of lumpy skin disease virus in cattle in the Poro Region of Ivory Coast
Source: Front Vet Sci. 2026 Mar 6;13:1759378. doi: 10.3389/fvets.2026.1759378 (PMC13002456; doi:10.3389/fvets.2026.1759378)
Supplement: Supplementary file 1 [file Supplementary_file_1.docx]

Supplementary Material

## Supplementary Figures


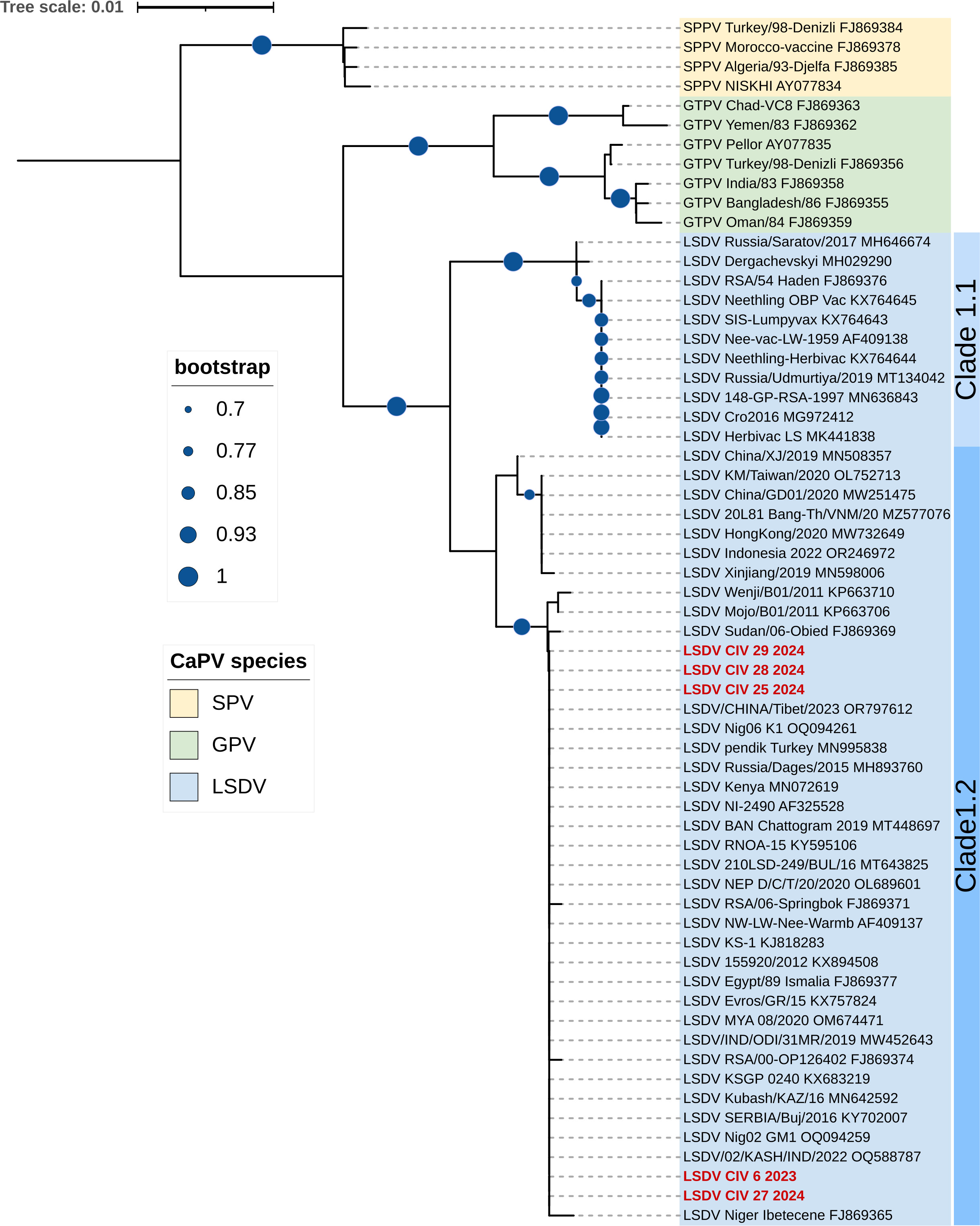


**Supplementary Figure S1:** Maximum-likelihood tree based on the complete GPCR gene sequences of CaPVs. The maximum-likelihood tree was constructed using the Tamura-Nei model with a Gamma distribution to illustrate the evolutionary relationships. LSDV from Ivory Coast are highlighted with red triangle


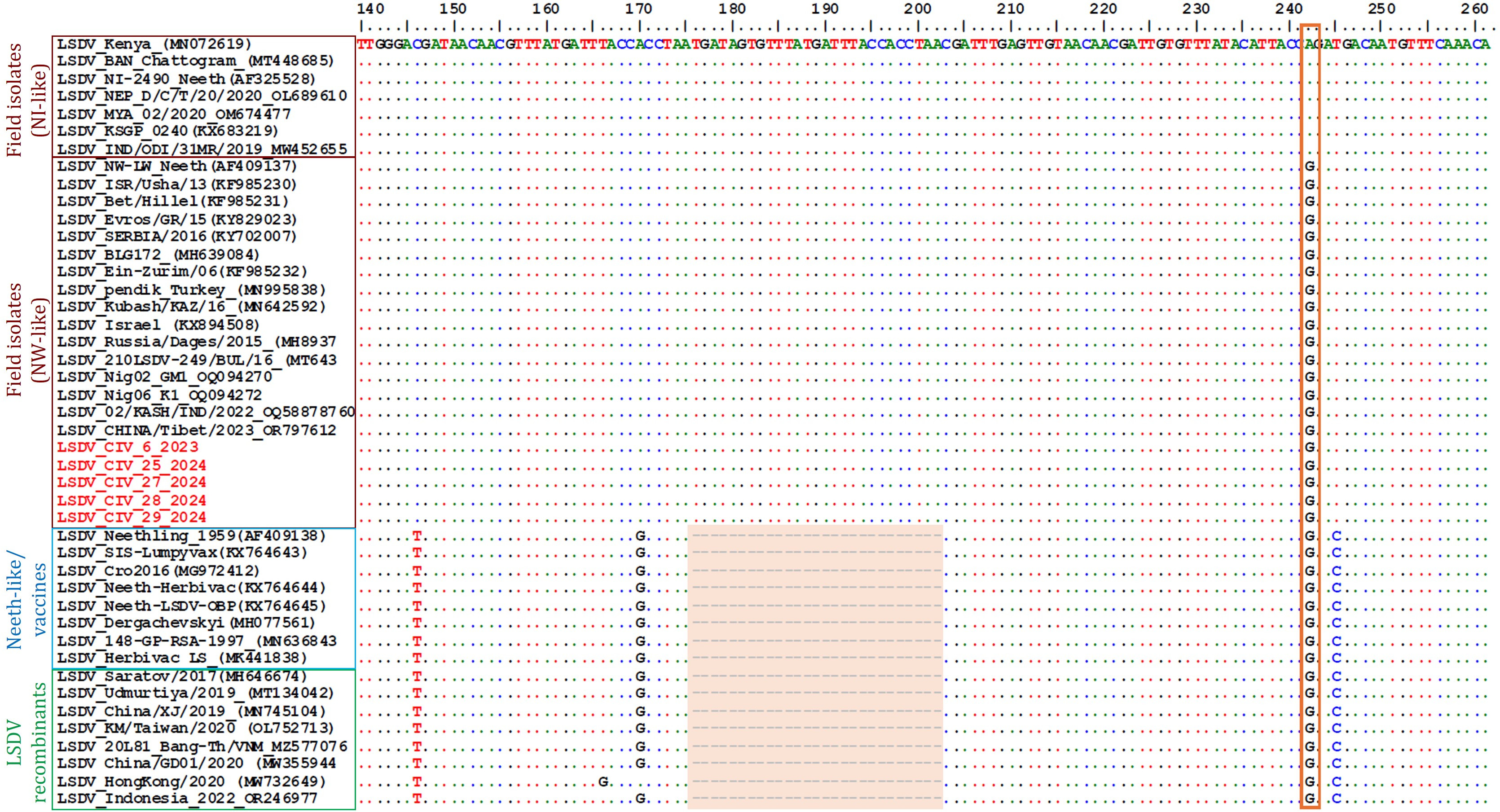


**Supplementary Figure S2:** Multiple sequence alignment of the partial nucleotide sequences of the EEV glycoprotein gene. The Ivory Coast isolates (in red) were aligned with representative LSDV sequences retrieved from GenBank. A 27-nucleotide deletion that is absent is highlighted in the box. The dots indicate the identical nucleotides in the alignment.
